# Application of Genetic Testing for Anorexia Nervosa: An Ethical Analysis

**DOI:** 10.1002/brb3.70406

**Published:** 2025-03-09

**Authors:** Sarah Ramsay, Kendra Allison, Heide S. Temples, Sara Sarasua, Luigi Boccuto

**Affiliations:** ^1^ Healthcare Genetics Program, School of Nursing Clemson University Clemson South Carolina USA

**Keywords:** anorexia nervosa, ethics, genetic testing, principlist, severe enduring anorexia nervosa

## Abstract

**Objective:**

Anorexia Nervosa (AN) is a severe, debilitating disorder with a high mortality rate. Research indicates that genetics plays a significant role in AN manifestation and persistence. Genetic testing has the potential to transform how AN is treated, however, in clinical practice, care must be taken to consider the ethical complexities involved. Our objective was to perform an ethical analysis of genetic testing in AN.

**Methods:**

We applied the principlist approach, taking into consideration the stakeholders involved and the core ethical principles of autonomy, beneficence, non‐maleficence, and justice to (1) evaluate the possible ethical implications of the use of genetic testing in the treatment of patients with AN, and (2) assess whether such testing is justified and if so, under what conditions.

**Results:**

Potential benefits of genetic testing identified include reduction of misdiagnosis and identification of treatable concurrent genetic conditions. The identified potential risks of genetic testing for possible AN‐associated risk variants outside of a research setting, especially without more effective treatment options, include a false sense of reassurance for those testing negative and a reduced emphasis on the importance of behavioral‐based therapies that may be of benefit.

**Discussion:**

Genetic testing for complex disorders, including AN, has tremendous potential, but is still primarily research‐based. Currently, for those presenting with atypical AN, and severe and enduring AN who, by definition, have not benefited from traditional treatment, genetic testing to rule out or identify other genetic conditions could be of benefit.

## Graphical abstract

Predictive and prescriptive genetic testing for complex disorders, including anorexia nervosa, has tremendous potential but is still primarily research‐based.

We performed an ethical analysis of the use of genetic testing in the treatment of anorexia nervosa.

Based on this analysis we propose that for those presenting with atypical anorexia nervosa and severe and enduring anorexia nervosa who have not benefited from traditional treatment, genetic testing to rule out or identify other genetic conditions may be of benefit.

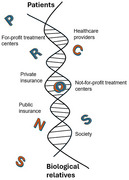

## 1 Introduction

Anorexia nervosa (AN) is a severe, debilitating, and often misunderstood disorder (Bemporad 1996). Although there is a lack of consensus as to what defines recovery from AN, it is estimated that approximately 20% of individuals with the disorder go on to develop a more intractable and treatment‐resistant form of the illness, often referred to as severe enduring anorexia nervosa (Eddy et al. 2017). Unfortunately, a significant number also die. The standardized mortality ratio (SMR) is the ratio of the number of deaths observed in a study population over a given period of time, versus the expected deaths in the population of origin (Kelsey 2008). An SMR greater than 1.0 indicates excessive deaths in the study population; with an SMR estimated to be between 5.9 and 15.9, AN is considered one of the deadliest psychiatric illnesses (van Eeden et al. 2021; van Hoeken and Hoek 2020).

Currently, there are no objective biomarkers capable of diagnosing AN, identifying those at risk for developing AN, or predicting its progression. There are no medications approved for the treatment of AN, and though valuable evidence‐based treatments including family‐based therapy and cognitive behavioral therapy have been developed, holistic treatment of AN, especially of those manifesting a severe and enduring phenotype, has made little progress. Anorexia nervosa relapse rates continue to be high, at least in part due to the significant expense of treatment, a dearth of investment in AN research, and a lack of focus on development of innovative and effective therapies (Guarda et al. 2018).

A considerable body of evidence indicates that genetics plays a significant role in AN etiology (de Jorge Martínez et al. 2022). Heritability (*h* ^2^_family,_ h^2^_twin_) estimates the degree to which a given phenotype can be attributed to genetic variation. Family and twin studies indicate heritability of 28%–83% for AN, with studies using narrower definitions and inclusion criteria consistently yielding higher estimates (de Jorge Martínez et al. 2022; Bulik et al. 2019; Dellava et al. 2011; Kortegaard et al. 2001; Steiger and Booij 2020; Wade et al. 2000).

Genetic testing has the potential for use as a tool to identify those at risk of developing AN and to diagnose and tailor patient‐specific treatment for those currently manifesting symptoms. This in turn could reduce the cost burden for healthcare systems and improve the quality of life for those who continue to struggle. Though routine genetic testing is not currently suggested for AN, it has been endorsed by several organizations for neurodevelopmental disorders, including autism spectrum disorder (ASD) (Manning and Hudgins 2010; Schaefer and Mendelsohn 2013; Siddharth et al. 2019), the ethical implications of which have been recently addressed (Morris et al. 2022). It is plausible that a similar endorsement of genetic testing may be put forth by leading eating disorder organizations in the future for AN.

The potential benefits of genetic testing come with significant ethical challenges that deserve consideration before implementing its widespread use in psychiatry as a whole and for AN in particular. With this in mind, we used the principlist approach to evaluate whether genetic testing for AN is ethically justified as well as under what conditions it may be in the future.

## 2 Materials and Methods

Principlism is the four‐principle approach to medical decision‐making developed by Beauchamp and Childress (Beauchamp 2007). The four principles are (1) respect for autonomy (individuals’ freedom and choice), (2) nonmaleficence (not harming others), (3) beneficence (doing good for others), and (4) justice. One must also identify the stakeholders involved. For the purposes of this assessment, principlism's well‐established framework allows for a structured evaluation of the possible benefits, risks, and overall utility of genetic testing. Legislation governing the age of consent for treatment, testing, and participation in research varies globally (Mathews 2023). Here we focus on older adolescents and adults consenting for minor children and legal frameworks in the United States.

## 3 Results: Ethical Analysis of Genetic Testing for AN Using the Principlist Approach

### 3.1 Overview of Relevant Literature

#### 3.1.1 Genetics of AN and the Use of Genetic Testing

Several AN‐specific Genome‐Wide Association Studies (GWAS) have been performed in the past two decades. Earlier studies suffered from technical limitations and were underpowered, thus their results have been questioned (Boraska et al. 2014; Nakabayashi et al. 2009; Wang et al. 2011). The Psychiatric Genome Consortium (PGC) has conducted two GWAS studies on AN. The first comprised 3495 individuals with AN and 10,982 controls and identified one locus on chromosome 12 with genome‐wide significance (rs4622308, *p* = 4.3 × 10^−9^). Loci in the specified region have also been associated with Type 1 diabetes, rheumatoid arthritis, and other autoimmune conditions (Duncan et al. 2017). The second study, conducted by Watson et al. (Watson et al. 2019), included 16,992 individuals with AN and 55,525 controls, identified eight significant loci (*p *< 0.05), and supported the first study's suggestion that AN be reconceptualized as a metabo‐psychiatric disorder. These findings are promising and suggest that as larger studies are conducted, more variants of significance will be found, as has been the case for schizophrenia and ASD (Dennison et al. 2020; Searles Quick et al. 2021).

Genetic variants, also termed mutations, are differences in the genetic sequence in the genome and can be benign, pathogenic, or of unknown significance (Tan et al. 2015). Certain disorders, including cystic fibrosis, sickle‐cell anemia, and phenylketonuria, are caused by variants in a single gene (Chial 2008). These so‐called single gene disorders can be dominant or recessive, and autosomal, sex‐linked, or mitochondrial. In contrast, complex disorders, including psychiatric disorders, are influenced by multiple genetic variants combined with environmental influences (International Society of Psychiatric Genetics 2019). GWAS are used to identify genomic variants associated with complex disorders. Polygenic risk scores (PRSs) use data generated from GWAS and are calculated from the sum of total weighted risks imparted by variants for a disorder. PRSs provide an estimate of the risk that an individual has of developing complex disorders based on their genetic makeup (Zhai et al. 2023). GWAS are powerful and valuable tools, however, they typically focus on the additive impact of common SNPs (minor allele frequency, or MAF > 0.05) (Bomba et al. 2017) and more often than not, for complex non‐mendelian diseases like AN, the identified SNPs explain only a small portion of heritability. The remaining “missing heritability” can at least in part be attributed to high‐impact rare variants (MAF < 0.01). In addition, GWAS often uses a binary, case‐control approach, as opposed to a quantitative trait association design. Though psychiatric illnesses, including AN, are typically diagnosed in a binary yes/no manner, in reality, they exist on a continuum (Lindeman et al. 2001; Tomczyk et al. 2023). It may well be that using a quantitative trait association study design with participants manifesting the extremes of the AN phenotype could uncover significant rare variants (Goswami et al. 2021). A recent comprehensive review of the genetic contribution to psychiatric disorders found that the ratio of SNP‐based heritability to heritability (*h* ^2^_SNP_/*h* ^2^), a measure of the contribution of common variants to AN, was only 27% (Baselmans et al. 2021). Moreover, though a study of genome‐wide variants in AN found no rare or low‐frequency (0.01 < MAF < 0.05) variants of significance, this may be due to the use of a case‐control rather than quantitative trait association design (Huckins et al. 2018).

Importantly, GWAS, PRS, and similar analyses are based on our current knowledge base and encompass only part of the genetic contribution to risk. In turn, genetics contributes only part of the overall risk for the development of a complex disorder. Unlike single gene disorders, genetic testing by itself is not diagnostic for complex disorders and must be combined with other risk assessments (Murray et al. 2021; Hoop et al. 2008).

#### 3.1.2 Genetic Testing in Comparable Neurodevelopmental Disorders

AN is often comorbid with other psychiatric and neurodevelopmental disorders, and several studies have demonstrated extensive genetic overlap among these disorders (Ihm et al. 2023; Lu et al. 2024; Grotzinger et al. 2022). It is understood that the offspring of those with psychiatric and neurodevelopmental disorders are at higher risk of developing not only the parental condition but various others as well (Martel et al. 2017). Moreover, AN should be considered during the investigation of the family history of individuals with psychiatric and neurodevelopmental disorders falling into the same genetic network. 2024Findings from neurodevelopmental disorders, such as ASD and schizophrenia, may provide important insights for AN. Heritability of ASD has been estimated at 50%, with an etiologic contribution from genetics around 90%, making it similar to AN in these respects (Bourgeron 2016; Silver and Rapin 2012). Genetic testing has been shown to help identify those with ASD risk factors that may be treatable and to aid in medication selection. Recently Kreiman and Boles published a comprehensive guide for clinicians considering incorporating genetic testing as part of ASD therapy (Kreiman and Boles 2020). The guide provides an excellent overview and is accessible to healthcare professionals with varying levels of genetic literacy. It presents the risks, benefits, and limitations of testing using examples from clinical practice. A similar guide may be useful for clinicians treating AN.

Though genetic testing and counseling for neurodevelopmental and psychiatric disorders have the potential to be helpful, they are still underutilized in practice (Hippman et al. 2016; Hunter et al. 2010; Inglis et al. 2015; Moldovan et al. 2017). Genetic testing for children diagnosed with ASD is recommended by the American College of Medical Genetics and Genomics, the American Academy of Neurology, and the American Academy of Pediatrics. However, though several studies have found parents open to the idea, awareness and utilization still remain low (Genovese and Butler 2020; Zebolsky et al. 2020; Zhao et al. 2021). For example, Zebolsky et al. found that in a survey of ASD support group members, only 53.6% of caregivers for children with ASD were aware that genetic testing exists for ASD and only 17.4% had completed testing (Zebolsky et al. 2020). The reasons for this are complex and include cost and inequitable access, uncertainty in the interpretation of findings, and a lack of genetic literacy among clinicians, patients, and caregivers (Austin 2020).

Schizophrenia is another psychiatric disorder with a complex environmental and genetic etiology. Heritability has been estimated at 81% for schizophrenia, making genetics the single‐most significant risk factor for its development (Sullivan et al. 2003). Certain types of genetic testing, including copy number variant analysis, have been suggested as useful complementary tools in schizophrenia treatment (Chen et al. 2021). As schizophrenia can be grouped with neurodevelopmental disorders, genetic testing may be helpful in cases with overlapping symptoms. However, there is no consensus suggesting that testing be done routinely until diagnostic yields improve (Savatt and Myers 2021).

#### 3.1.3 Genetic Testing for AN

Genetic testing outside of the research environment has yet to be endorsed for the diagnosis or treatment of patients with AN by professional organizations, though it is being used in various treatment settings (Lutter 2023). GWASs have been able to identify clear differences between AN and other eating disorders, but studies have not evaluated genetic differences between restricting and binge/purge subtypes of AN, emphasizing the need for the inclusion of more detailed behavioral information in research studies (Hübel et al. 2021). AN risk variants have been correlated with those for body mass index (BMI), fat mass and fat‐free mass, cholesterol, and fasting insulin, suggesting a metabolic contribution to the disorder (Watson et al. 2019). Genetic testing for metabolic variants may prove uniquely beneficial to better tailor refeeding and treatment plans for those with AN (Duncan et al. 2017; Watson et al. 2019). The immune system is intricately linked with AN (Sirufo et al. 2022). Inflammatory bowel disease (IBD) and other digestive disorders share many symptoms with AN, making it difficult for clinicians to delineate which came first, especially given that refeeding during AN treatment is almost always accompanied by gastrointestinal discomfort (Mascolo et al. 2017). Genetic testing to identify gastrointestinal disorder‐associated variants may help with this delineation (Wu et al. 2019).

Medical complications associated with AN contribute to its propensity for misdiagnosis (Puckett et al. 2021). As discussed in the analysis, we identified numerous case study reports of individuals being misdiagnosed with AN when in fact they had other genetic disorders or of those with AN who had concurrent genetic disorders that had gone undiagnosed (Dalle Grave et al. 2022; Feeney and Buell 2018; Khatri et al. 2022; Takeuchi et al. 2015).

#### 3.1.4 Stigmatization and Historical Context

Though stigmatization, shame, and discrimination are common with all mental illnesses, the additional perception that AN is voluntary or even a lifestyle choice, is somewhat unique (Crisafulli et al. 2008). Few studies have evaluated patient perceptions of genetic risk, testing, and counseling among those with AN. Using online surveys of those with eating disorders, Michael et al., found a lack of understanding of genetic risks to offspring (Michael et al. 2020). However, after being exposed to hypothetical genetic counseling situations, participants reported decreased feelings of guilt, shame, and stigmatization, suggesting that future use of genetic counseling in the eating disorder population may be useful.

### 3.2 Ethical Analysis

#### 3.2.1 Stakeholders

Genetic testing has potential implications not only for patients but also for the patients’ families, healthcare practitioners, not‐for‐profit treatment programs, the for‐profit treatment industry, public and private health insurance providers, and society as a whole. Potential implications for these stakeholders are considered throughout the assessment and summarized in Table 1. The four principles are considered herein, taking into account the following possible outcomes of genetic testing for those with AN: (1) Identification of a risk variant for AN, (2) no AN risk variant being identified/variant of uncertain significance (VUS) identified, and (3) a risk variant for another genetic disease being identified. It is also necessary to consider these outcomes both in light of the limits of current knowledge and the possibility of future scientific findings and developments.

**TABLE 1:** Stakeholder Impact of genetic testing for AN.

Current knowledge base				
Stakeholder	Pros and cons of testing positive or negative for a genetic variant			
	Testing positive		Testing negative	
	Pros	Cons	Pros	Cons
**Patient**	For AN‐associated variants, it could reduce feelings of shame. For conditions with similar symptomology, it could provide alternative diagnoses and more effective treatments.	For AN‐associated variants, it could lead to feelings that their fate is sealed as no new treatment course would be taken. For conditions with similar symptomology, could lead to increased worry and stigmatization.	For AN‐associated variants, could lead to feelings of relief and increased optimism.	For AN‐associated variants, it could lead to feelings of confusion, anger, and a false sense that they are “in the clear”. Also, it could place emphasis on family dynamics and environment, leading to feelings of blame, guilt, and shame. For conditions with similar symptomology, could lead to feelings of hopelessness, as alternative treatment would not be available.
**Biological relatives**	For AN‐associated variants, could institute preventative measures for those not currently exhibiting symptoms. For other conditions, it could lead to awareness, treatment, and/or preventative actions.	For AN‐associated variants, could lead to decreased emphasis on important family/environmental impact.	For AN‐associated variants, or other conditions, could lead to feelings of relief and increased optimism.	For AN‐associated variants, could lead to over‐emphasis on family/environmental impact. Could result in reduced vigilance and ignoring symptoms if they do develop.
**Healthcare providers**	If testing positive for another condition, it could provide healthcare providers with more effective treatments. If testing positive for AN‐associated variants, could lead to over‐emphasis on biological basis without enough scientific support. If testing negative, it could lead to changing treatment direction without adequate scientific support to do so.			
**Not‐for‐profit treatment centers**	Testing in general can add to the knowledge base. If partnered with testing companies, could provide additional revenue for these under‐funded centers. However, as public insurance will most likely not pay, and patients are more likely to not be in a financial position to pay, could lead to additional costs to the program and/or inequitable access for patients.			
**For‐profit treatment centers**	If testing positive for AN‐associated variants, it could be used as an additional argument to insurers, patients, and families for the need of continued treatment. If partnering with testing companies, it could be another revenue source. If testing negative for AN‐associated variants or positive for another condition, it could detract from the argument that the patient needs to stay in treatment at the center, discharge of patient, and decreased revenue.			
**Private insurance**	May pay for testing for other conditions with similar symptomology. Could reduce treatment costs associated with those testing positive for another treatable condition. Alternatively, it could lead to increased costs if the other condition is chronic and expensive to treat. Will not pay for unapproved testing, including AN. If testing positive for AN‐associated variant, there is no cost reduction associated with the patient, possible reduction in costs for biological relatives if preventative actions are taken. In general, this could lead to additional expenses for unproven and unsupported treatments due to lack of more definitive scientific support. Any cost reduction or increase could be passed on to other insurance holders.			
**Public insurance**	See private insurance. Where cost savings or increases are indicated, this would also reduce or increase costs for taxpayers.			
**Society**	Testing in general could decrease societal stigmatization of AN by emphasizing the biological nature of the disease. However, it could also result in decreased support for programs aimed at reducing societal pressures such as over‐emphasis on physical appearance.			

Abbreviation: AN, anorexia nervosa.

#### 3.2.2 Autonomy

An individual's right to autonomy entails the ability to make decisions about their own health and well‐being. In order to make a truly autonomous choice, the action must be voluntary in nature. If the patient is being coerced or pressured to either have genetic testing or not, then the principle of autonomy is not met. Possible alternatives to testing should also be presented and the patient must be competent to make the decision (Huibers and van `t Spijker 1998). In the case of AN in general, respect for the patient's autonomy is often overridden by clinicians. This is based on the argument that the patient is not thinking clearly due to starvation, and thus not physically competent to consent to treatment (Thaler et al. 2016). Once a patient is medically stable, the argument is that the patient is not mentally competent (Tan et al. 2008). Attempting to obtain consent for genetic testing from those manifesting a severe and enduring AN phenotype could be considered inappropriate, at least for those regarded as being medically and/or mentally “unstable”. However, consent for lifesaving treatment and consent for genetic testing are very different scenarios. To avoid violating patient autonomy, if such testing is warranted, it is suggested that patients with AN should be assessed as any other patient, and accordingly, be considered competent to consent to genetic testing unless extreme psychological and/or physical impairment is clearly present. A similar argument has been made for adults with schizophrenia experiencing a psychotic episode consenting to treatment (Hostiuc et al. 2018). Taking the opportunity to consent or not consent to genetic testing away from the patient would be unethical.

The type of consent to use should also be considered. Though typically associated with research, proper consent for genetic testing in non‐research settings is critical. Genetic testing is unique in that later assessment of the same test data may uncover additional findings, especially in a rapidly developing space like psychiatric genetics. Numerous iterations of informed consent for genetic testing have been proposed, each with its own balance of pros and cons (Bunnik et al. 2021). A recent in‐depth survey and literature review by Ormond et al. provides a list of critical concepts for inclusion in consent, such as voluntariness, reasons for testing, limitations, and to whom the results should be reported (Ormond et al. 2021). Given how quickly genomic science is progressing and the fact that new variants will most likely be discovered, a meta‐consent model may be most appropriate. Meta‐consent allows the individual to dictate if, how, and when they would like to provide consent for the assessment of their genetic data in the future, thereby respecting patient autonomy and allowing patients to take a more active role in their care (Cumyn et al. 2021).

It is also essential to consider the autonomy of the patient's biological relatives. Genetic testing of the patient may violate the autonomy of their relatives if the testing reveals that the relative or the relative's offspring may be at risk when said relative may not have wanted to know. For example, if testing reveals a disease variant that may be inherited by or from family members. The “right not to know” is often overlooked in this increasingly data‐driven era but should be given equal consideration as the right to know (Berkman and Hull 2014). Considering the unique nature of genetic testing, the concept of relational autonomy, whereby a person's individual autonomy is considered in relation to their situation in a relational group, rather than individual autonomy alone, may be more suitable (Hostiuc et al. 2018). The appropriateness of disclosing patient personal protected information (PPI), in this case genetic test results, to at‐risk relatives is the subject of ongoing debate and is complicated by conflicting legal precedence (Clayton et al. 2019; Perry et al. 2020).

#### 3.2.3 Beneficence

The principle of beneficence requires that healthcare providers act in the best interest of their patients (Kinsinger 2009). While genetic testing for patients first diagnosed with AN could potentially identify individuals at risk of developing a more severe phenotype, no specific interventions or treatments currently exist for this situation, thus the treatment strategy would be no different than for patients without an identified genetic risk. However, it is possible that other treatable conditions with similar phenotypes could be identified through genetic testing. For example, citrin deficiency (CD), an autosomal recessive metabolic disorder caused by pathogenic variants of the *SLC25A13* gene, which encodes the mitochondrial transport protein citrin, mimics AN, and has been misdiagnosed as such (Takeuchi et al. 2015). Autoimmune Addison disease, a potentially fatal condition that presents with a lack of appetite, weight loss, and tiredness, and is associated with the HLA‐DRB1*04:04 variant of the *HLA‐DRB1* gene, has also been misdiagnosed as AN (Feeney and Buell 2018). McArdle disease, also known as glycogen storage disease Type V (GSDV), is an autosomal recessive genetic disorder caused by loss‐of‐function pathogenic variants of the *PYGM* gene with clinical features like those of AN. AN and GSDV have the potential to negatively synergize, and their similar clinical features could delay the diagnosis and treatment of either condition (Dalle Grave et al. 2022). Delay in diagnosis of AN can also be exacerbated by genetic testing revealing a condition with similar clinical features, as was recently reported in a case of Pseudo Bartter syndrome, a rare autosomal recessive tubulopathy with multigenetic etiology (de Alves Pereira Carvalho Saraiva et al. 2022). Thus, genetic testing to rule out other disorders and consideration of how other genetic disorders interact with AN, especially in those manifesting a more severe and enduring AN phenotype, would appear to be of benefit to the patient.

If we consider the patient as a part of a familial group, possible benefits may also extend to biological relatives. For example, a family member may discover that they too have a treatable genetic condition. Looking to a future where a more tangible genetic link is identified for AN, and better treatments are available, some evidence suggests that genetic testing and counseling could decrease feelings of stigma, shame, and guilt (Michael et al. 2020). Furthermore, for those whose genetics indicate a risk of developing AN, and for the disorder to become more severe and intractable, proactive preventative care may be of benefit. For example, ensuring that weight loss, regardless of the cause, is addressed promptly in those not yet exhibiting AN symptoms. However, these possible benefits must be weighed against potential harms resulting from information gleaned as a result of genetic testing.

#### 3.2.4 Non‐Maleficence

The principle of non‐maleficence states that healthcare providers must always attempt to ensure that the actions they take do not cause harm to their patients. In this respect, genetic testing for AN may have unintended negative consequences.

As outlined earlier, several potentially significant genetic variants have been identified in recent AN studies. However, further research is needed to confirm these findings. Furthermore, if these variants are confirmed to confer risk for AN, currently there is no indication that a different course of treatment than what is currently in place for a patient would or should be pursued. Thus, testing for these potential AN‐risk variants outside of a research setting may be of little use. However, we can consider the possibility that genetic testing may discover variants linked with disorders other than AN. First, if such a variant is discovered, as noted earlier, the individual could be treated for the associated condition. However, care must be taken in this situation to not overlook or ignore the comorbid AN, and the strength of the identity the patient may have developed with it over time (Croce et al. 2024). The egosyntonic nature of AN often manifests in the patient seeing it as protective and powerful, and the thought of no longer having that perceived protection or control can be extremely distressing (Gregertsen et al. 2017). The other identified genetic disorders may also have serious health implications as well as risks for stigmatization and discrimination. If such a disorder is not discovered, the patient may feel hopeless, especially if their current AN treatment program is inadequate, and no additional effective alternatives are available and accessible.

Looking to a future where a clearer link between specific variants and the development of AN is identified, testing positive for such variant(s) could result in the patient feeling that their fate is sealed and that further treatment is of little use. Considering the high suicide rate for those with AN, the risk for self‐harm could be elevated (Bulik et al. 2008). The patient and family may see the current state as permanent, and the patient's biological parents may have feelings of guilt that they “gave” their child the disorder. Psychiatric genetic counseling, regardless of whether it is paired with genetic testing, has been shown to be beneficial to patients and their families. Such counseling addresses the myths and realities of heritability in polygenic disorders which many clinicians are not familiar with (Austin 2020).

In situations where a variant associated with AN is not identified, the patient may feel defensive. Friends and family may also question the reality of the patient's illness. Patients and families may feel that genetics has been ruled out as a contributing factor and attribute cause to family dynamics, which can lead to feelings of guilt and blame (Wufong et al. 2019). Lastly, a negative result could provide a false sense of reassurance for at‐risk individuals not yet displaying symptoms, and signs and symptoms of AN may be ignored if they do manifest.

As previously noted, when discussing beneficence, evidence suggests that genetic testing may reduce the stigma associated with AN (Michael et al. 2020). However, the perception that genetic testing could increase stigmatization, especially by those who disagree that they have a mental illness, has also been expressed (Easter 2012). The possibility that genetic testing could hamper recovery by reducing the emphasis on personal responsibility in the recovery process, and the role that the environment plays, has also been articulated by those with AN and treatment professionals (Kong et al. 2017).

#### 3.2.5 Justice

In the healthcare arena, the principle of distributive justice proposes that resources should be distributed fairly and equitably (Varkey 2021). The concept of procedural justice holds that people should be treated with dignity, respect, consistency, and lack of bias and should have the opportunity to participate in decision‐making about their care (Prusiński 2023). Genetic testing can be expensive, though costs have decreased significantly in recent years. Between 2015 and 2022, the sequencing cost per Megabase (Mb) decreased from $0.04 to $0.006 and per genome sequencing costs went from $3970 to $525 (Wetterstrand 2023). Payment for such testing in the United States comes from private insurance, Medicaid, Medicare, or out‐of‐pocket funds. At present, no professional organization nor the United States Food and Drug Administration (FDA) recommends the use of predictive or prescriptive testing for AN, which decreases the likelihood of coverage by public and private health insurance. Thus, given the cost and the fact that payment would be limited to those who can afford the out‐of‐pocket expense, equal access to testing would be challenging. However, for those who can afford to pay out of pocket for testing, their choice to do so should not be limited by the positions taken by regulatory agencies and professional organizations. Each situation is unique and must be considered individually.

In a future scenario, where knowledge has progressed and such testing is widely supported for AN, the question becomes whether it is cost‐effective. In the predictive realm, given the substantial and ongoing expense of treating AN, the cost of genetic testing appears justified if it helps identify those at risk and preemptive actions are taken. The same could be said for prescriptive genetic testing, but only if more effective treatments than are currently available exist.

Discrimination and stigmatization of those with mental illness are common, and testing positive for a variant predisposing someone to AN could result in discrimination. For employers, the individual may be considered not just a financial risk, but a legal liability in the workplace, especially in a healthcare setting (Boyd et al. 2016; Brower 2021). To thwart the potential injustice of discrimination based on genotype, the Genetic Information Nondiscrimination Act (GINA) was enacted in 2008 (122 Stat. 881 2008). However, stigmatization and implicit bias are challenging to control from a legal standpoint. Nevertheless, there is hope that the “geneticization” of mental illness will decrease its associated stigma and discrimination by demonstrating its biological nature (Corrigan and Watson 2004).

#### 3.2.6 Genetic Testing Options

With the ethical assessment outlined above as background, the following options for types of genetic testing are presented for consideration. Table 1 provides a summary assessment of stakeholder impact.

1. Testing to rule out other genetic disorders with phenotypes similar to AN.

For those presenting with atypical AN and for those with severe and enduring AN who have not had success with current best practice treatments, genetic testing to determine whether another condition is at play may be warranted. If testing indicates that another condition is involved, it is critical that the patient's treatment team prepare a therapeutic transition plan.

2. Testing for variants that research has shown may be associated with AN.

Given the current lack of consensus on which variants are most pertinent and the lack of therapeutic options for those testing positive for said variants, testing outside of a research setting for possible AN‐associated risk variants is not currently supported. However, given the need for research participants, it is suggested that patients be presented with the opportunity to participate in genetic studies, especially those with a severe and enduring AN phenotype and their biological relatives, with or without AN. Guidelines for approaching those with eating disorders for participation in research have been developed by the Eating Disorders Genetic Initiative (EDGI) and recently expanded to better ensure the inclusivity of a wide range of participants (MacDermod et al. 2022).

In a future scenario where there is better alignment on relevant genetic variants, for those who may be at risk for the development of AN and those with AN at risk of progressing to a more severe and enduring phenotype, it is suggested that genetic testing in a clinical setting should be proposed if there is a clear therapeutic path available to those who test positive or negative for AN‐risk alleles.

3. Pharmacogenetic testing.

As stated earlier, no drugs are approved for the treatment of AN. Nonetheless, clinicians commonly employ pharmacotherapy in its treatment, often for weight gain (Frank 2020). Medications may also be of benefit in the treatment of comorbid conditions. An in‐depth review of the use of pharmacogenetics in psychiatry is beyond the scope of this paper. However, the FDA issued a statement in 2019 reiterating that the majority of such testing has not been FDA‐approved (US Food and Drug Administration 2019). That same year, the International Society of Psychiatric Genetics (ISPG) issued an updated statement about the use of pharmacogenetics in psychiatry. The Society recommended that such testing “should be viewed as a decision‐support tool to assist in the thoughtful implementation of good clinical care”. However, they also state that “when pharmacogenetic testing results are already available, providers are encouraged to integrate this information into their medication selection and dosing decisions” (International Society of Psychiatric Genetics 2019).

For the purposes of this article, it is suggested that pharmacogenetic testing be offered where there is significant evidence to indicate that it may be of benefit as a complimentary tool in the prescription and dosing of certain medications such as anticonvulsants, antipsychotics, and mood stabilizers (Lunenburg and Gasse 2020; van Schaik et al. 2020).

## 4 Conclusion

Genetic and genomic testing is progressing rapidly and understanding genetics’ role in the development and persistence of AN is becoming increasingly important. Such testing has the potential to have a significant impact on how those with AN are treated and in turn could reduce the morbidity and mortality rates for the disorder. However, the successful translation of research findings into clinical practice requires careful consideration of the ethical implications involved.

Given that specific predictive and diagnostic variants have not yet been identified for AN, the ethical risk‐benefit balance for genetic testing for proposed AN‐risk variants outside of a research setting suggests not testing. However, testing for variants associated with genetic conditions with a clinical presentation similar to AN may be warranted, especially for those with a severe and enduring AN phenotype. In a future scenario where additional support for AN‐significant loci, and better treatment options for those testing positive or negative are available, genetic testing for those with AN has the potential to decrease stigmatization and revolutionize how this often‐misunderstood and misdiagnosed disease is treated.

Considering the current knowledge base, some may argue that this type of ethical assessment is premature. We propose that such analyses are timely, that significant efforts to increase genetic and genomic literacy in the healthcare community are needed, and that guidelines and best practices should be developed proactively to ensure that the healthcare community is adequately prepared to provide their patients with the care, education, and support they need both now and as the science evolves.

## Author Contributions

**Sarah Ramsay**: conceptualization, research, writing–original draft. **Kendra Allison**: review and editing. **Heide S. Temples**: review and editing. **Sara Sarasua**: review and editing. **Luigi Boccuto**: review and editing.

## Conflicts of Interest

The authors declare no conflicts of interest.

### Peer Review

The peer review history for this article is available at https://publons.com/publon/10.1002/brb3.70406.

## Data Availability

Data sharing not applicable to this article as no datasets were generated or analyzed during the current study.
